# Medical Society

**Published:** 1897-11-27

**Authors:** 


### MEDICAL SOCIETY.

### The Treatment of Pyo-pneumothorax.

There has long been much discussion as to the proper method of treatment in cases of pyo-pneumothorax. Partly, no doubt, this has arisen from the fact that, while in some cases it is itself the main disease, in others it is but the final accident by which death is brought about in old-standing disease of the lung, so that it becomes difficult to lay down any general rules as to treatment which shall be applicable in all cases.

Dr. Samuel West, in the paper which he read to the Medical Society last Monday, advocated the extended use of free incisions in its treatment. Putting the matter into few words, he would treat pyo-pneumothorax on the same principles and in accordance with the same indications as apply to empyema.

Various reasons have been adduced against this course, and the very striking and interesting case related by Dr. West, a case which had been under several physicians, and had been practically condemned to be let alone, was sufficient to prove how widely spread is the opinion that to open the chest in such cases is but to court disaster. However, it was opened, and in the end it did perfectly well,

In regard to the assertion often made that in these cases the lung is so tied down by adhesions and thickening of the pleura that its expansion is impossible, Dr. West urged that the evidence of this was to a large extent the evidence of the post-mortem room, and that in many of the cases observed this binding down of the lung would not have occurred if operative interference had been undertaken earlier. As a fact, in the case mentioned the lung rapidly expanded after operation, filling up the whole chest.

Whether this is likely to occur in a majority of cases is a point which will no doubt be much disputed. Even Dr. West admits that this must depend much on the period at which the disease comes under treatment. Again, the question of the persistence of the perforation of the lung, by which the pyo-pneumothorax was produced, is one which must have some bearing on the problem, while undoubtedly there will always remain a residuum of cases in which, as we have already said, pyo-pneumothorax is but the final accident in a chronic disease, and in which operative interference is out of the question. We may well agree, however, with Dr. West that the fact of there being air in the pleura should in no way be held to stand in the way of operation, and that many such cases may properly be treated as he suggests, and it certainly is satisfactory to hear on several hands a protest raised against the doctrine that effusion has a beneficial influence in checking the development of tuberculosis, a doctrine which has even quite lately received high support, such pleurisies being spoken of as "providential."

In the discussion several matters of interest came up, the most important having reference to the position of the patient during and after operation, the great point being not to interfere with the respiration of the sound side. Cases were mentioned in which sudden death had taken place on turning the patient on to his sound side, from a sudden invasion of the lung by pus from the diseased pleura; and in regard to after treatment it was said to be desirable not to apply any bandages to the chest, but to allow the discharge, which was often very profuse, to run into a considerable bedding of wood wool sprinkled with iodoform, which could be removed and replaced by the nurse without disturbing the patient.

